# Improving Interlayer Adhesion of Poly(p-phenylene terephthalamide) (PPTA)/Ultra-high-molecular-weight Polyethylene (UHMWPE) Laminates Prepared by Plasma Treatment and Hot Pressing Technique

**DOI:** 10.3390/polym13162600

**Published:** 2021-08-05

**Authors:** Long Zhu, Dmitriy A. Dikin, Simona Percec, Fei Ren

**Affiliations:** 1 Department of Mechanical Engineering, Temple University, Philadelphia, PA 19122, USA; long.zhu@temple.edu (L.Z.); ddikin@temple.edu (D.A.D.); 2 Temple Materials Institute, Temple University, Philadelphia, PA 19122, USA; simona.percec@temple.edu

**Keywords:** poly(p-phenylene terephthalamide), ultra-high-molecular-weight polyethylene, plasma treatment, interlamellar adhesion

## Abstract

Poly(p-phenylene terephthalamide) (PPTA) is a high-performance polymer that has been utilized in a range of applications. Although PPTA fibers are widely used in various composite materials, laminar structures consisting of PPTA and ultra-high-molecular-weight polyethylene (UHMWPE), are less reported. The difficulty in making such composite structures is in part due to the weakness of the interface formed between these two polymers. In this study, a layered structure was produced from PPTA fabrics and UHMWPE films via hot pressing. To improve the interlayer adhesion, oxygen plasma was used to treat the PPTA and the UHMWPE surfaces prior to lamination. It has been found that while plasma treatment on the UHMWPE surface brought about a moderate increase in interlayer adhesion (up to 14%), significant enhancement was achieved on the samples fabricated with plasma treated PPTA (up to 91%). It has been assumed that both surface roughening and the introduction of functional groups contributed to this improvement.

## 1. Introduction

Poly(p-phenylene terephthalamide) (PPTA) is a high-performance polymer with high toughness, high tensile strength, and chemical stability at high temperatures. PPTA fibers and fabrics have been widely utilized as reinforcement in composite materials [1,2,3,4]. To construct PPTA-reinforced composites, thermosetting resins, such as epoxies and phenolics, are usually used as the matrix to bond fibers or fabrics [5,6,7]. Sometimes, micro and nano sized fillers, such as silica, SiC, graphite, ZnO, and carbon nanofiber, are introduced in the composites to further improve their performance [8,9]. 

Ultra-high molecular weight polyethylene (UHMWPE) is another high-performance polymer. Due to its good mechanical, chemical, and biocompatible properties, it has gained great popularity in many applications including medical devices, body armors, marine infrastructure, and automobiles [10,11,12]. Various forms of UHMWPE, such as powders, fibers, yarns, fabrics, and laminated sheets are used as the reinforcement phase to fabricate composites where the matrix materials ranged from polyurethane [13,14,15], low-density polyethylene (LDPE) adhesive film, to epoxies [16,17]. 

There have been some efforts to produce PPTA/UHMWPE composites. For example, Hofsté et al. prepared composites from chopped PPTA fibers and UHMWPE powder [18]. The authors found that the mechanical properties of the resulting composites were not as good as the theoretically predicted values, which was attributed to the void content in the composite as well as the weak adhesion between the UHMWPE matrix and the PPTA fibers [18]. In another study, Hofste et al. used chromic acid to chemically oxidize the UHMWPE powder before mixing it with the PPTA fiber [19]. They found that the wear resistance and other mechanical properties were improved due to stronger fiber/matrix adhesion. However, the oxidized surfaces were unstable, which degraded over time [19]. Li and coauthors also fabricated PPTA/UHMWPE composites from PPTA fibers and UHMWPE powder [20]. They used silane, which contains both hydrophobic and hydrophilic groups, to enhance the interfacial bonding between the PPTA fiber and the UHMWPE matrix. The authors pointed out that the fiber ends within the composites were the limiting factors of the mechanical properties. Packing defects and Poisson contraction were other key factors impacting the performance of the composite. However, the authors did not provide information on the interfacial strength. In a recent study, Guleria et al. developed a microwave-assisted compression molding process for the fabrication of PPTA/UHMWPE composites [21]. Although the authors claimed that the microwave heating process could improve the mechanical properties, including ultimate tensile strength, impact energy absorption, flexural and hardness properties, this study only compared UHMWPE/Kevlar^®^ composite with pure UHMWPE samples. 

In addition to infusing PPTA fibers into UHMWPE powder-based matrix, knitting PPTA and UHMWPE yarns is another method to fabricate PPTA/UHMWPE composites [22,23]. Adhesive resins, such as polyurethane, vinyl ester [24] and ethylene vinyl acetate [25] could also be used to enhance the bonding between the components in laminated composites containing PPTA fabrics and UHMWPE fabrics/fibers. It can be seen that in this type of composite structure, the UHMWPE and the PPTA materials do not form direct bonds with each other. Moreover, the additional resin components involved additional processing steps, such as solution preparation, sample impregnation, mixing, and various post treatments (heating, curing, drying, etc.). In other developments, reinforcements, such as carbon nanotubes, were introduced in the resin matrix and the authors claimed that the carbon nanotubes enhanced the energy absorption capability by fracturing, bridging, and pull-out mechanism [26].

As above-mentioned, the weak interfacial bonding was found to adversely affect the performance of the composites [27,28,29]. For PPTA fibers and fabrics, the chemical stability and the smoothness of their surfaces make it difficult to achieve good adhesion between them and other parts of the composite [30,31]. Therefore, it is necessary to treat these surfaces in order to increase the bonding strength. Various surface modification methods have been developed to improve the bonding strength of composite materials [10], such as acid treatment [32,33], chemical modification [34,35], radiation-induced grafting [36,37,38], and plasma treatment [39,40,41]. 

Among these methods, plasma treatment is of particular interest because of: (i) its effectiveness in surface modification without changing the bulk properties of the processed materials; (ii) ease of implementation; and (iii) minimal environmental impact (no acids or organic solvents are needed) [42]. There are four major effects of plasma treatment on the surface: (a) cleaning (b) etching, (c) chemical modification, and (d) crosslinking of near-surface molecules [43]. Surface modification by plasma processes has been well documented in the literature. For example, Su et al. studied the effect of oxygen plasma treatment on Kevlar^®^ fibers and Kevlar^®^ fiber/bismaleimide composites. They found that the oxygen–plasma treatment could change the chemistry and the morphology of the surface, which in turn improved the water resistance, dielectric properties, and interlaminar shear strength (40% higher than that of the untreated) [42]. Park and coworkers examined the interfacial adhesion of oxygen–plasma treated poly(p-phenylene-2,6-benzobisoxazole (PBO, Zylon) and Kevlar^®^ fibers/epoxy composites using micromechanical techniques and surface wettability test. They attributed the increased interfacial shear strength (14.8% higher for PBO composite and 16% higher for Kevlar^®^ composite in comparison to those without treatment) and work of adhesion (13.5% higher for PBO composite and 11.7% higher for Kevlar^®^ composite in comparison to those without treatment) to induced hydrogen bonding and covalent bonding as a result of plasma treatment [44]. Guo et al. found that tribological performance of the prepared Kevlar^®^ fabric/phenolic composites could be improved with plasma treatment by introducing nitrogenous and oxygenic groups as well as roughening of the surface [30]. 

As previously discussed, the majority of the efforts to make PPTA/UHMWPE composites were focused on PPTA fibers and UHMWPE powders. There is limited information in the open literature on PPTA/UHMWPE laminates made with continuous UHMWPE film material. In this study, we report the research on producing a laminar structure consisting of PPTA fabrics and UHMWPE films using a facile hot-pressing protocol. Oxygen plasma treatment was applied to both materials and their surface characteristics, both topological and chemical, were altered. Our results showed that the interlaminar adhesion could be enhanced by treating either the PPTA or the UHMWPE surfaces. However, significant improvement was achieved when the plasma treatment was conducted on the PPTA fabrics. With the controllable interlaminar adhesion, future work will focus on PPTA/UHMWPE composite panels, targeting body armor applications. 

## 2. Experimental Section

### 2.1. Materials

The PPTA fabrics (area density: 218 g/m^2^, thickness: 0.37 mm, weave: plain) woven from Kevlar^®^ 49 fiber and UHMWPE film (thickness: 0.2 mm) were purchased from Goodfellow Corp. (Coraopolis, PA, USA). The epoxy glue (JB weld epoxy) was purchased from JB Weld Company (Sulfur Springs, TX, USA).

### 2.2. Oxygen Plasma Treatment

The UHMWPE film cut into pieces (1” × 1.5”) was sonicated with acetone, rinsed with deionized water, and dried with nitrogen. Oxygen plasma treatment of UHMWPE and PPTA fabrics (1.5” × 2”) was performed using a Harrick Plasma cleaner PDC-32G (3” diameter × 6.5” length Pyrex chamber) (Harrick Plasma Inc., Ithaca, NY, USA) for different times. Surface free energies of UHMWPE with different plasma power and treatment time were calculated from contact angle measurements (Table S1). A plasma power of 18 W was used in this study since it resulted in the highest surface free energy.

### 2.3. Preparation of PPTA/UHMWPE/PPTA Laminate 

**Scheme 1:**
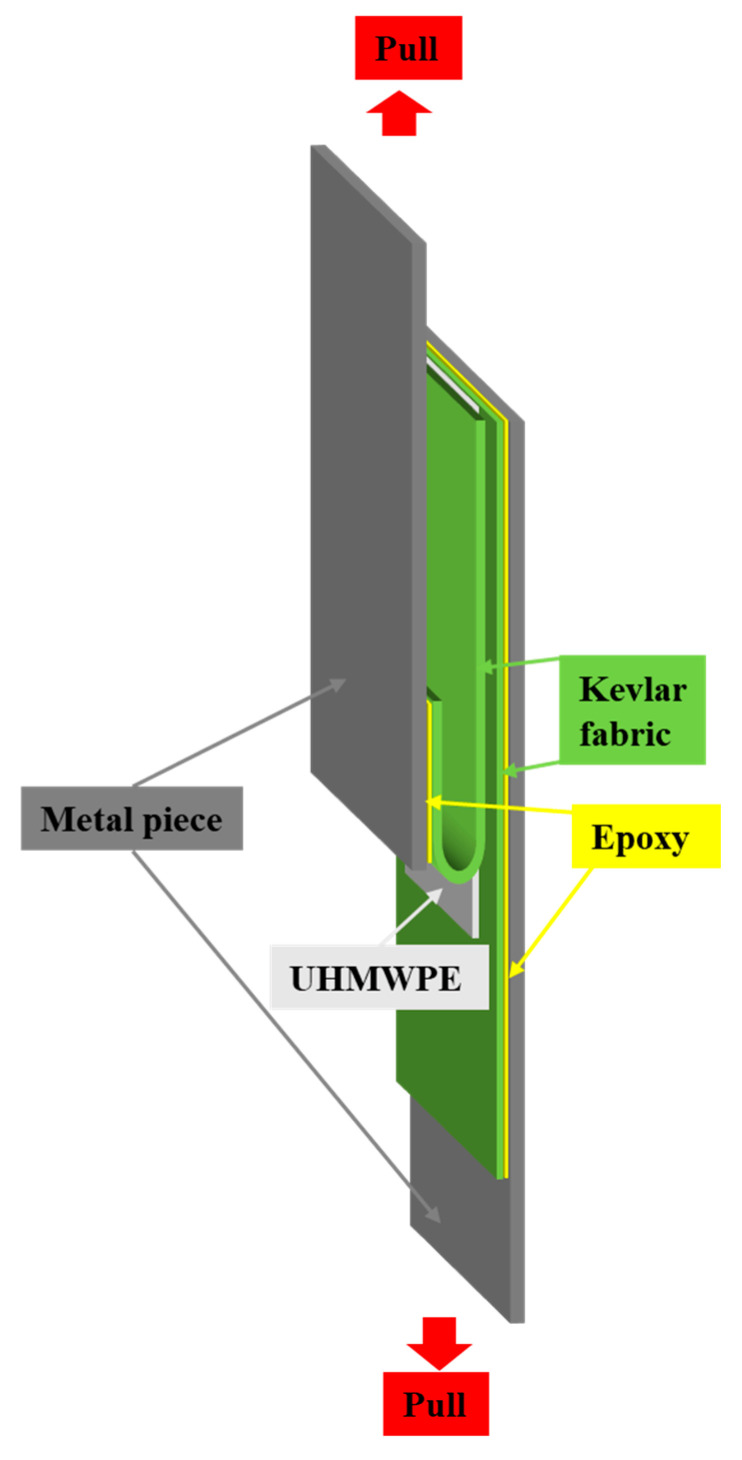
Schematic showing sample configuration for peeling test.

Heat press machine used in preparation of laminate was purchased from Across International, Inc. (Livingston, NJ, USA). Two pieces of PPTA fabrics and one piece of UHMWPE film were stacked to a laminate structure with the UHMWPE film in between, as shown in Scheme 1. The UHMWPE film was placed in the center with respect to the edges of the PPTA pieces and the edges of the UHMWPE were carefully aligned with the fiber direction in the PPTA fabric. The pressure was adjusted through the force adjusting knob on the top of the hot-pressing machine, which was calibrated in advance by using a thin film force sensor (FlexiForce sensor, Tekscan Inc., Boston, MA, USA). For all samples, the pressure was controlled to be about 73.9 kPa during the hot pressing.

**Figure 1:**
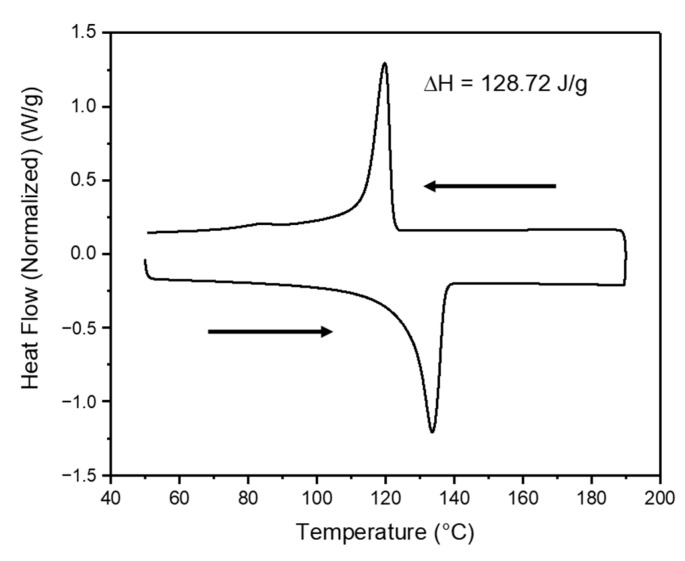
Differential Scanning Calorimetry heat flow curve of UHMWPE film at a heating rate 4.25 °C/min, dwell time 1 h, and cool down at 3.5 °C/min rate.

Differential scanning calorimetry (DSC) was used to study the thermal behavior of the UHMWPE film (Figure 1), whose melting point was determined to be 134 °C. Our previous work showed that the following processing parameter would yield composite samples with good mechanical properties without significant oxidation: heating rate = 4.25 °C/min, peak temperature = 190 °C, holding time = 1 h, and cooling rate = 3.5 °C/min.

### 2.4. 180-Degree Peel Test

The peel test was conducted according to ASTM D903-98 using a universal testing machine (H5K-T, Tinius Olsen Inc., Horsham, PA, USA). Sample configuration is shown in Scheme 1. The aluminum pieces were cleaned with acetone and deionized water prior to sample fabrication. Two PPTA pieces on the outer surface were glued on the aluminum pieces using epoxy with UHMWPE film as the interlayer. After the epoxy was fully cured at room temperature in air for 24 h, the sample was installed on the testing machine with two aluminum pieces clamped onto the grips of machine. The peeling test was performed with a speed (peeling rate) of 25.4 mm per minute. The peeling force versus extension curve was recorded. At least six samples were tested for each condition and the peeling forces were averaged to calculate interlamellar adhesion strength for comparison [45].

### 2.5. Characterization of PPTA Fabrics and UHMWPE

The surface morphologies of PPTA fabric and UHMWPE film after plasma treatment were characterized using scanning electron microscopy (SEM, Quanta 450 FEG, Thermo Fisher-FEI, Hillsboro, OR, USA), atomic force microscopy (AFM, Dimension Icon, Bruker, Billerica, MA, USA). The surface chemical group were analyzed by Fourier transform infrared spectroscopy (FTIR, Thermo Fisher Scientific, Waltham, MA, USA) and X-ray photoelectron spectroscopy (XPS, VersaProbe 5000, Physical Electronics Inc., Chanhassen, MN, USA). For XPS, a 100 μm monochromatic Al Kα (1486.6 eV) X-ray beam was used to irradiate the sample surface. The C1s peak was calibrated to 285.0 eV and CasaXPS was utilized to carry out peak fitting procedure. Failure analysis was conducted by examining the surface morphologies after peeling test with SEM.

## 3. Results and Discussion

### 3.1. Morphologies of PPTA Fabric and UHMWPE Film

**Figure 2:**
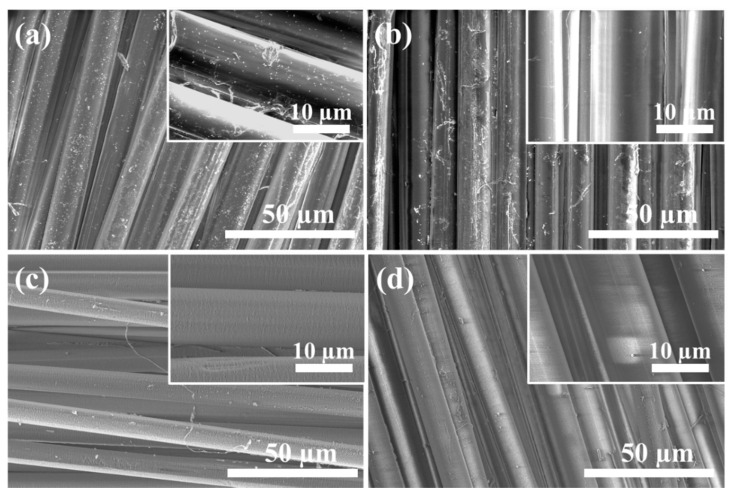
SEM images of PPTA fabric without treatment (**a**) and with oxygen plasma treatment for 1 min (**b**), 5 min (**c**) and 10 min (**d**). The insets show magnified images.

The morphology of the PPTA fabric is shown in Figure 2. The untreated PPTA fabric (Figure 2a) showed a flat surface with some debris left on the surface (inset of Figure 2a), likely as a result of previous processing or handling. When PPTA fabrics were treated with plasma for 1 min, there was still a large amount of debris on the surface, but small particles were removed (inset of Figure 2b), indicating some degree of cleaning by the plasma. In contrast, the PPTA sample with 5 min plasma treatment showed a clean surface without debris (Figure 2c). However, an uneven nanotextured surface with appearance of pitting holes and trenches developed, likely as a result of the etching effect. The increased porosity of the outmost layer of PPTA fibers can effectively increase the contact area between the fabric and the molten UHMWPE penetrating these pores, and thus facilitate mechanical interlocking and bond strength between layers [46]. When the plasma treatment time was extended to 10 min, the outer layer of the fiber became smoother again with only some visible etching debris on it (Figure 2d). This does not facilitate the interlocking between the layers and the bonding strength between the UHMWPE and PPTA fabric surface weakens.

**Figure 3:**
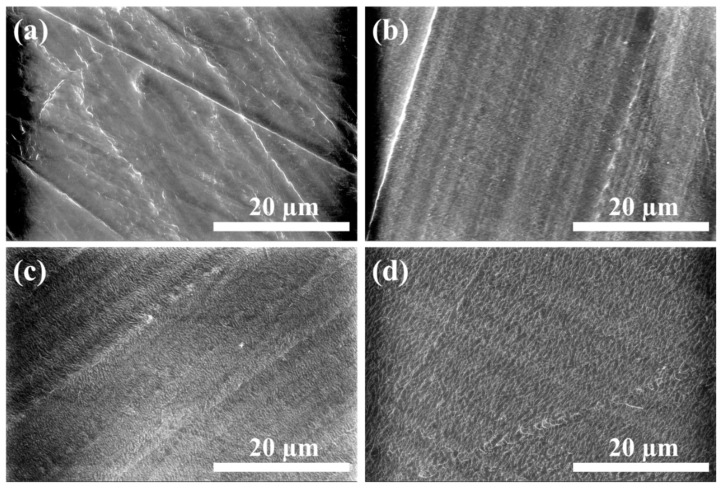
SEM images of UHMWPE film without treatment (**a**), with oxygen plasma treatment for 1 min (**b**), 2 min (**c**) and 5 min (**d**).

Figure 3 shows the morphologies of UHMWPE film with different plasma treatment times. Untreated UHMWPE exhibited randomly oriented, deep, but smooth grooves (Figure 3a), most likely as a result of the polymer film manufacturing process. When the surface was plasma-treated, nanotextured roughness appears and initially existing grooves are smoothed out (Figure 3b–d), an effect similar to that observed on plasma-treated UHMWPE fibers reported by Lee et al. [46]. With the increase in plasma treatment time, the pores are expanded, and the grooves almost disappear (Figure 3d).

**Figure 4:**
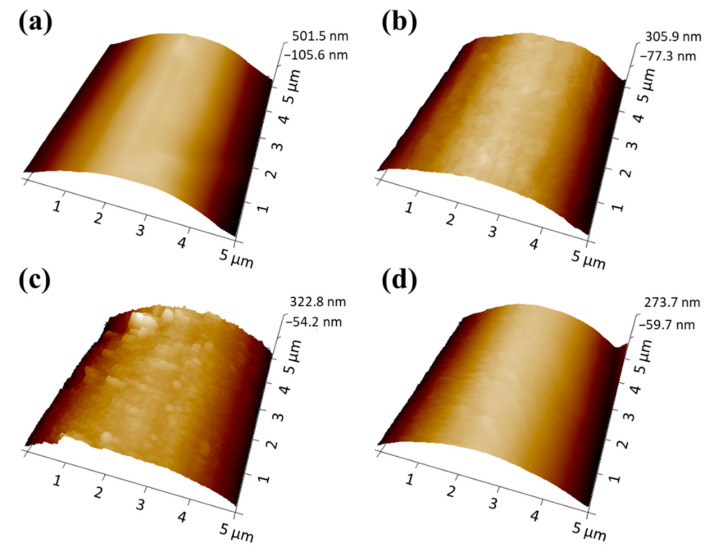
AFM images of PPTA fiber before plasma treatment (**a**), with plasma treatment for 1 min (**b**), 5 min (**c**) and 10 min (**d**).

**Figure 5:**
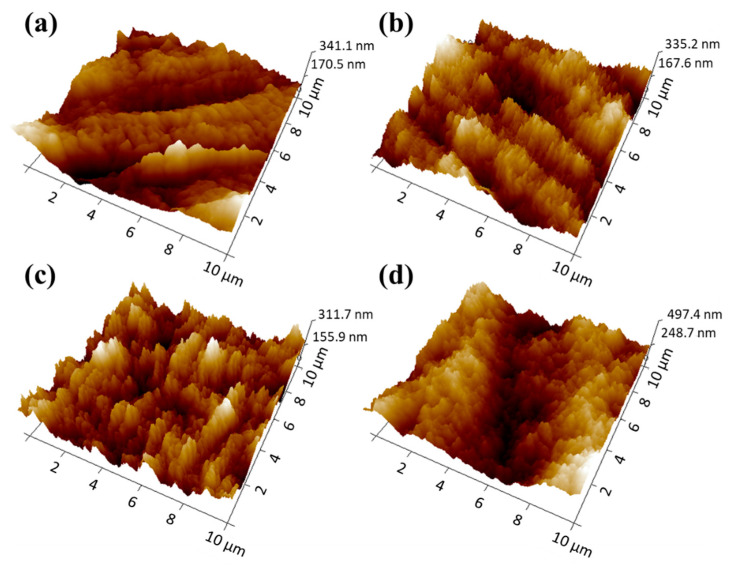
AFM images of UHMWPE before plasma treatment (**a**), with plasma treatment for 1 min (**b**), 2 min (**c**) and 5 min (**d**).

AFM analysis was further carried out to examine the surface morphologies of both PPTA fabric and UHMWPE film as a result of the plasma treatment (Figure 4 and Figure 5). The untreated PPTA showed a smooth surface (Figure 4a), whereas some granular like structures were observed on the plasma-treated PPTA samples (Figure 4b–d). The size of this granular like structure initially increased when the treatment time increased from 1 min to 5 min (Figure 4b,c), but then significantly reduced when the treatment was extended to 10 min (Figure 4d). This trend agrees with the SEM observation where extended plasma treatment would result in over etching (Figure 2d).

The AFM results of UHMWPE with different treatment times are shown in Figure 5. Deep grooves and large granular like structures were detected on the surface of the untreated sample (Figure 5a). After being treated for 1 min and 2 min, fine acicular structures or trenches were created on the surface (Figure 5b,c). When exposed to the longer treatment time (5 min), the grooves and the fine acicular structures were etched, leaving micro-pits on the surface (Figure 5d).

### 3.2. Surface Chemical Analysis of PPTA and UHMWPE Samples

#### 3.2.1. FTIR

**Figure 6:**
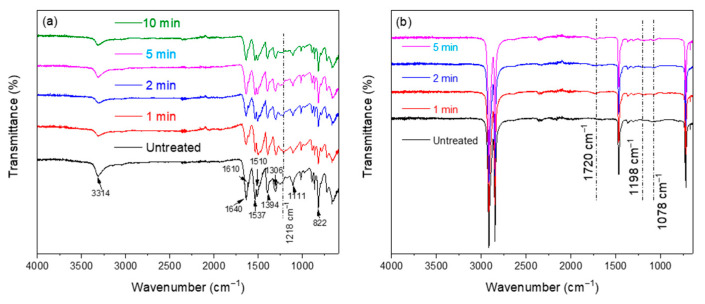
FTIR spectra of PPTA (**a**) and UHMWPE (**b**) treated for different times.

FTIR was performed to investigate possible chemical changes induced by the plasma treatment. Figure 6a exhibits the FTIR spectra of the PPTA samples, where several major absorption peaks for PPTA structure can be detected. The peaks at 3314 cm^−1^ and 1537 cm^−1^ correspond to the stretching vibration and the bending vibration of the N–H bonds, respectively. The peaks at 1394 cm^−1^ and 1111 cm^−1^ correspond to the stretching vibration of C–N. Peaks at 1610 cm^−1^ and 1510 cm^−1^ correspond to the stretching vibration of C=C. The stretching vibration of C=O in amide carbonyls and the bending vibration of C–H result in two peaks at 1640 cm^−1^ and 822 cm^−1^, respectively [47]. The peaks at 1306 cm^−1^ and 1300–1255 cm^−1^ can be ascribed to C–N stretching and the symmetrical deformation of the –NO_2_ group [48]. On closer examination, a peak unrelated to PPTA structure is observed at 1218 cm^−1^. This peak corresponds to the stretching mode of oxygen containing group C–O, which may be introduced by the plasma treatment. 

Figure 6b shows the FTIR spectra of UHMWPE samples with different treatment times. Four major peaks of UHMWPE are observed at 2913 cm^−1^, 2850 cm^−1^, 1473 cm^−1^ and 719 cm^−1^, which correspond to the non-symmetric stretching vibration, the symmetric stretching, the bending vibration, and the in-plane vibration of –CH_2_– bond, respectively [49]. Two small peaks were observed at 1720 cm^−1^ and 1198 cm^−1^, which correspond to the C=O stretching and C–O stretching, respectively. The peak at 1078 cm^−1^ related to the C–O stretching mode became stronger after plasma treatment. The emergence of these oxygen containing bonds is believed to be a result of the plasma treatment. 

Since plasma treatment only modifies the topmost surface (nanometer scale) while the FTIR detection depth is usually on the level of micrometers [50], the relative intensity of the oxygen-containing groups on the top surface is weak. 

#### 3.2.2. XPS

**Figure 7:**
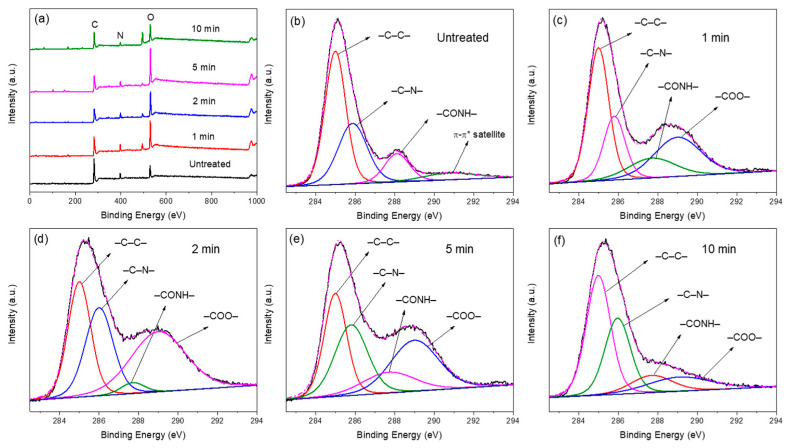
XPS spectra of (**a**) PPTA fabrics with different plasma treatment times. Deconvoluted C1s peaks of (**b**) untreated PPTA and PPTA treated for (**c**) 1 min, (**d**) 2 min, (**e**) 5 min, and (**f**) 10 min.

In addition to FTIR, the X-ray photoelectron spectroscopy (XPS) technique was also used to examine the surface chemistry of both the PPTA fabric and the UHMWPE film samples. Figure 7a compares the full-scan spectra of the PPTA samples before and after plasma treatment. Three peaks around 285 eV, 400 eV and 532 eV are observed, which correspond to the three main elements, C, N, and O, in the PPTA molecule. Table 1 summarizes the chemical composition of PPTA samples with various plasma treatment times (untreated, 1 min, 2 min, 5 min and 10 min). It was found that after 1 min of plasma treatment, the percentage of oxygen increased to 27.83% in comparison to the untreated sample where the oxygen was only 13.10%. The oxygen concentration further increased in the 5 min plasma treated sample. However, the oxygen percentage decreased in the 10 min treated sample, which may be a result of the etching effect caused by the extended plasma treatment that removed the functional groups introduced at the initial stage of the treatment process [42]. 

The content of carbon, however, showed an opposite trend, with a decrease from 80.05% (untreated) to 63.71% (1 min plasma treatment). The deconvoluted C1s peaks of untreated PPTA and those treated for 1 min, 2 min, 5 min and 10 min are compared in Figure 7b–f. The C1s peak of the untreated PPTA (Figure 7b) consists of contributions from four carbon-containing functional groups, which are –C–C– at 285 eV, –C–N– at 285.8 eV, –C=O at 288.1 eV, and π-π* shakeup satellite at 290.8 eV [51]. For the plasma-treated PPTA samples (Figure 7c–f), the C1s peaks can be deconvoluted to –C–C–, –C–N–, –C=O and –COO– peaks, whose binding energies are 285 eV, 285.8 eV, 287.7 eV, and 289.0 eV, respectively. It can be seen that the plasma treatment process introduced a large amount of the –COO– group (Table 1), which may contribute to the enhanced binding between PPTA and UHMWPE via covalent or hydrogen bonds. However, when the treatment time was increased to 10 min, the percentage of the –COO– group decreased, likely due to the etching effect of the longer treatment time. 

**Figure 8:**
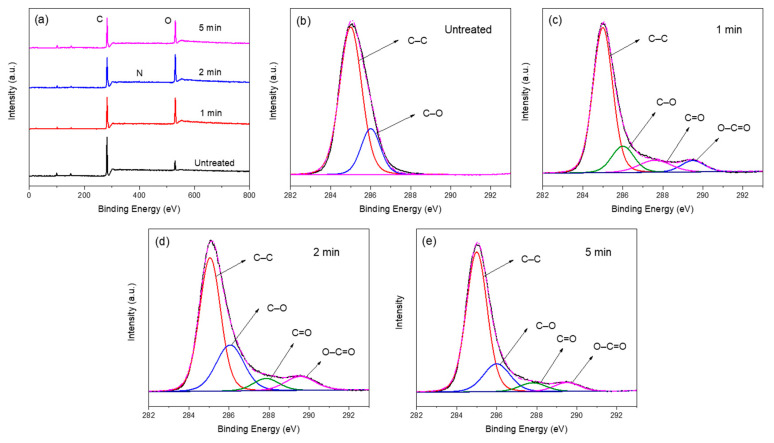
XPS spectra of (**a**) UHMWPE films with different plasma treatment time. C1s core level spectra of UHMWPE samples (**b**) without plasma treatment, and after plasma treatment for (**c**) 1 min, (**d**) 2 min and (**e**) 5 min.

The XPS results of UHMWPE films with different plasma treatment times are shown in Figure 8. As can be seen from the full-scan spectra in Figure 8a, all samples showed the C1s (285 eV) and the O1s (530 eV) peaks. In addition, a very weak N1s peak (400 eV) was found in the sample with 2 min plasma treatment. As a result of the plasma treatment, the intensity of the C1s peak decreased, whereas the intensity of the O1s peak increased dramatically, indicating the introduction of oxygen-containing groups. The quantitative chemical analysis result is summarized in Table 2. The O/C atomic ratio increased from 0.08 to 0.28 after 1 min of plasma treatment, which further increased to 0.30 after 2 min, followed by a decrease to 0.28 when the plasma treatment time was extended to 5 min. This reverse trend may be due to the etching effect of the plasma. Figure 8b–e compare the C1s core-level spectra of the UHMWPE samples with various plasma treatments (untreated, 1 min, 2 min and 5 min). The C1s peak of the untreated UHMWPE was deconvoluted into C–C (285 eV) and C–O (286 eV) bonds. In plasma-treated UHMWPE samples, two additional oxygen-containing groups (C=O at 288 eV and O–C=O at 289.5 eV) were found. The percentages of these functional groups in different samples were summarized in Table 2. The results showed that after 2 min of plasma treatment, the content of C–C group decreased from 78.51% to 58.32%, whereas that of the oxygen-containing groups increased, with substantial increases in the C=O and O–C=O groups. Again, when the treatment time increased to 5 min, the percentage of these oxygen-containing functional groups dropped, likely owing to the etching effect.

### 3.3. Adhesion Properties 

**Figure 9:**
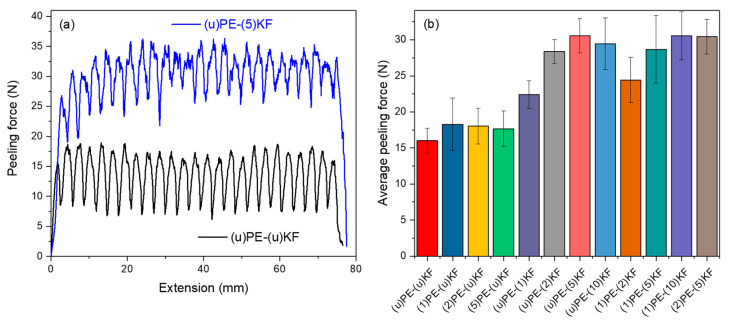
Peel-off force versus extension curves for a sample without treatment and a sample with PPTA treated for 5 min (**a**); and average peel-off force for different samples (**b**). PE: UHMWPE film. KF: PPTA fabric. U: untreated. The numbers in the brackets indicate treatment time in minutes.

Figure 9a exhibits two representative relationships between force and extension for a 180-degree peel test. The peaks and valleys in the curves correspond to the geometrical distances between the fiber strands in the PPTA fabrics. Since the peak values on the curves represent the forces needed to separate the UHMWPE and the PPTA layers, the average of all peaks was defined as the peeling force. A stronger peeling force was observed for the sample with treated PPTA, indicating a higher adhesion strength. 

The average peeling forces for all samples included in this study were summarized in Figure 9b. In general, when plasma treatment was only performed on UHMWPE, the composite samples showed a mediocre increase in the peeling force (up to 14.3%), as compared to the samples without plasma treatment. Additionally, increasing treatment time on UHMWPE did not lead to a further improvement in adhesion. In contrast, significant enhancement, as high as 91.1%, was observed when the PPTA fabrics were plasma-treated. This can be explained as follows. When PPTA was treated, chemical functional groups and rough surface textures were being introduced on the cleaned surface. First of all, the surface textures can increase the contact area as the molten UHMWPE penetrates the PPTA surface microcracks and which can enhance mechanical interlocking. Secondly, the active oxygen-containing functional groups were expected to interact with the molten UHMWPE during hot pressing and form strong bonds between UHMWPE film and PPTA fabric layers, based on chemical reaction theory [43]. It is well-known that plasma treatment can enhance surface energy and wettability, which also facilitates the above processes. However, when only UHMWPE was plasma treated, the debris or particles left on the surface of PPTA could weaken the bonding or mechanical interlocking of the penetrated molten UHMWPE, since these loose debris or particles, as a barrier, preclude the physical and chemical interaction between PPTA and molten UHMWPE. Another possible reason for this behavior could be that the effect of plasma treatment on UHMWPE was erased in the molten state at high temperature and pressure during hot press procedure, thus the treatment of UHMWPE contributed little to increasing adhesion.

The treatment time of the PPTA fabrics had a significant effect on the improvement of adhesion. As shown in Figure 9b, for samples with only PPTA treated, the peeling force gradually increased when the treatment time increased from 1 min to 5 min, then dropped when the treatment time increased to 10 min. This indicates that there is an optimum required time for the plasma treatment process [30,44]. When the plasma treatment time is shorter than the optimum treatment time (e.g., <5 min in our conditions), its effect on surface modification, including etching, cleaning, and surface functionalization, will be insufficient. However, an extended treatment time will have opposite effects, which are caused by over-etching. As shown in Figure 2 and Figure 7f and Table 1, when the treatment increased to 10 min, the generated texture on the surface was removed and functional groups decreased. The degradation of fabrics could also occur due to longer treatment time [46]. 

### 3.4. Failure Analysis

**Figure 10:**
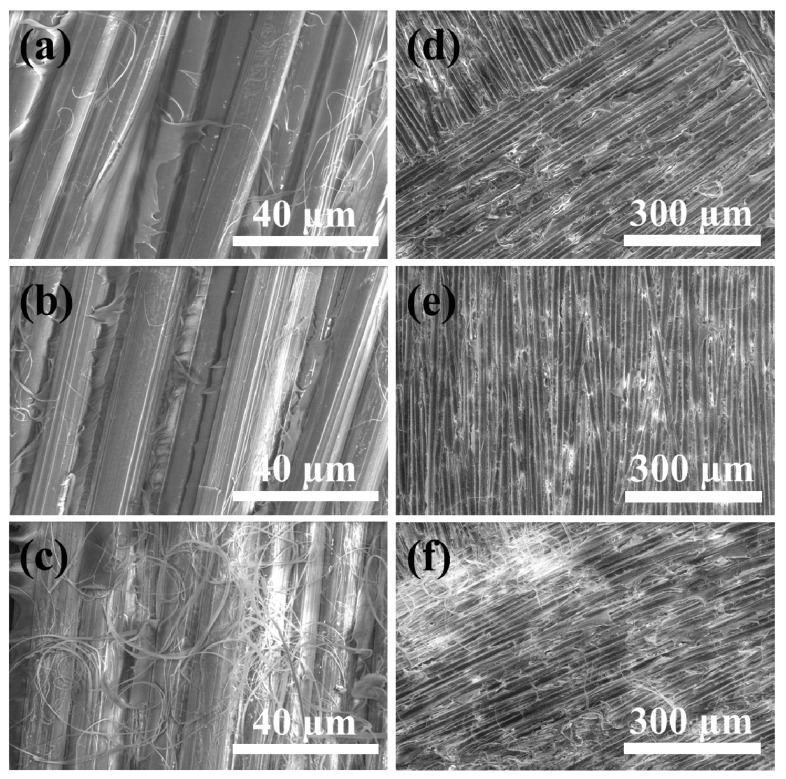
SEM images of PPTA fabric after peel test without plasma treatment (**a**); with only UHMWPE treated for 1 min (**b**); with only PPTA treated for 5 min (**c**) and SEM images of UHMWPE after peeling test without treatment (**d**); with only UHMWPE treated for 1 min (**e**); with only PPTA treated for 5 min (**f**).

The morphology of both PPTA and UHMWPE surfaces after the peel-off test is shown in Figure 10. The PPTA side is shown in Figure 10a–c, while the UHMWPE side is shown in Figure 10d–f. For the sample without any plasma treatment, the PPTA side showed a relatively clean surface (Figure 10a), which was likely created because of delamination between PPTA and UHMWPE, implying weak bonding between the two materials. When plasma treatment was conducted on the UHPWME, indications of a stronger interface were observed, such as broken fibrils from the PPTA fibers and left-over fractured UHMWPE on the PPTA side (Figure 10b). When the treatment was performed on the PPTA fabric, a large number of peeled off fibrils were observed (Figure 10c,f). This result indicated that the adhesion between PPTA and UHMWPE was high, and a significant portion of the failures occurred on the PPTA side. This fractographic result agrees with the trend obtained from the above-mentioned peel test. 

## 4. Conclusions

Laminate samples were fabricated from PPTA fabrics and UHMWPE films by hot pressing method. Oxygen plasma treatment was utilized to enhance the interlamellar adhesion between PPTA fabrics and UHMWPE film. SEM and AFM results showed that plasma treatment could modify the surface texture, while FTIR and XPS analyses demonstrated the surface functionalization induced by the plasma treatment. The 180-degree peel test result showed that the adhesion of the PPTA/UHMWPE laminate could be significantly enhanced when the PPTA fabrics were treated with the oxygen plasma. It was likely that the process of oxygen plasma treatment introduced active functional groups and cleaned and roughened the fiber surface, thereby improving the bond between UHMWPE and modified PPTA fabrics. PPTA/UHMWPE composites with controllable interlaminar adhesion for body armor applications will be studied in future work.

## Figures and Tables

**Table 1:** Surface chemical composition and functional groups of PPTA fabrics oxygen plasma treated at different times based on the XPS deconvoluted C1s peaks.

Treatment Time (min)	Chemical Composition (%)			Atom Ratio		Functional Groups (%)			
	C1s	N1s	O1s	O/C	N/C	–C–C–	–C–N–	–C=O	–COO–
**Untreated**	80.05	6.85	13.10	0.16	0.09	49.64	31.35	12.57	0
**1**	63.71	8.47	27.83	0.43	0.13	40.10	19.84	13.49	26.57
**2**	63.12	7.84	29.04	0.46	0.12	30.66	28.24	3.50	38.06
**5**	59.66	6.85	33.49	0.56	0.11	28.44	28.14	18.40	25.03
**10**	69.48	5.77	24.75	0.36	0.08	45.06	30.15	10.98	13.81

**Table 2:** Surface chemical composition and functional groups of UHMWPE films treated for different times based on the deconvolution of C1s peaks.

Treatment Time (min)	Chemical Composition (%)			Atom Ratio	Functional Groups (%)			
	C1s	O1s	N1s	O/C	C–C	C–O	C=O	O–C=O
**Untreated**	92.73	7.27	0	0.08	78.51	21.49	0	0
**1**	77.97	22.03	0	0.28	69.78	14.32	9.42	6.47
**2**	76.02	23.08	0.90	0.30	58.32	26.64	6.50	8.54
**5**	78.24	21.76	0	0.28	70.78	18.11	5.64	5.47

## Data Availability

Not applicable.

## Supplementary Materials

The following are available online at https://www.mdpi.com/article/10.3390/polym13162600/s1, Table S1: Surface free energy of UHMWPE films treated for different times and powers.
