# Supplementary material for: Improving Interlayer Adhesion of Poly(p-phenylene terephthalamide) (PPTA)/Ultra-high-molecular-weight Polyethylene (UHMWPE) Laminates Prepared by Plasma Treatment and Hot Pressing Technique
Source: Polymers (Basel). 2021 Aug 5;13(16):2600. doi: 10.3390/polym13162600 (PMC8400389; doi:10.3390/polym13162600)
Supplement: Supplementary file 1 [file polymers-13-02600-s001.zip › polymers-1325017-supplementary.pdf]

polymers

Supporting information

Improving Interlayer Adhesion of Poly(p-phenylene terephthalamide)(PPTA)/Ultra-high-molecular-weight Polyethylene (UHMWPE) Laminates Prepared by Plasma Treatment and Hot Pressing Technique

Long Zhu [1], Dmitriy A. Dikin [1], Simona Percec [2], Fei Ren [1,*]

[1] Department of Mechanical Engineering, Temple University, Philadelphia, PA 19122, USA; long.zhu@temple.edu (L.Z.); ddikin@temple.edu (D.A.D.); renfei@temple.edu (F.R.)
[2] Temple Materials Institute, Temple University, Philadelphia, PA 19122, USA; simona.percec@temple.edu
* Correspondence: renfei@temple.edu

Table S1. Surface free energy of UHMWPE films treated for different times and powers.

Treatment time (min)	Treatment power (W)	Average contact angle of water (°)	Average contact angle of glycerol (°)	Surface free energy (mJ/m²)
**Untreated**		95.63	83.93	21.05
**1**	**6.8**	86.39	82.51	20.89
**1**	**10.5**	40.40	45.35	58.72
**1**	**18**	37.11	41.90	60.86
**2**	**18**	36.90	35.32	59.2
**5**	**18**	26.10	25.96	55.19
